# Review of T-2307, an Investigational Agent That Causes Collapse of Fungal Mitochondrial Membrane Potential

**DOI:** 10.3390/jof7020130

**Published:** 2021-02-11

**Authors:** Nathan P. Wiederhold

**Affiliations:** Fungus Testing Laboratory, Department of Pathology and Laboratory Medicine, University of Texas Health Science Center, San Antonio, TX 78229, USA; wiederholdn@uthscsa.edu

**Keywords:** T-2307, aromatic diamidine, in vitro susceptibility, mitochondrial membrane, mitochondrial membrane potential, *Candida*, *Cryptococcus*

## Abstract

Invasive infections caused by *Candida* that are resistant to clinically available antifungals are of increasing concern. Increasing rates of fluconazole resistance in non-*albicans Candida* species have been documented in multiple countries on several continents. This situation has been further exacerbated over the last several years by *Candida auris*, as isolates of this emerging pathogen that are often resistant to multiple antifungals. T-2307 is an aromatic diamidine currently in development for the treatment of invasive fungal infections. This agent has been shown to selectively cause the collapse of the mitochondrial membrane potential in yeasts when compared to mammalian cells. In vitro activity has been demonstrated against *Candida* species, including *C. albicans*, *C. glabrata*, and *C. auris* strains, which are resistant to azole and echinocandin antifungals. Activity has also been reported against *Cryptococcus* species, and this has translated into in vivo efficacy in experimental models of invasive candidiasis and cryptococcosis. However, little is known regarding the clinical efficacy and safety of this agent, as published data from studies involving humans are not currently available.

## 1. Introduction

Infections caused by fungi are of increasing clinical concern. These include invasive diseases caused by strains of *Candida* species that are resistant to clinically available antifungals. As highlighted by the World Health Organization global surveillance report on antimicrobial resistance, resistance in non-*albicans Candida* species to fluconazole has been reported at rates of 30% or higher in multiple countries on several continents [1]. At some centers in the United States, increased rates of nonsusceptibility to the echinocandins have also been reported in *C. glabrata* [2,3]. Interestingly, some studies have also noted coresistance to both the echinocandins and the azoles in *C. glabrata* isolates [3,4]. Recently, the problem of antifungal resistance has been highlighted by the emergence of *Candida auris*, as many strains of this clinically significant pathogen are multidrug-resistant [5], and pan-antifungal resistance has also been reported [6,7]. In addition, the utility of clinically available antifungals is often limited by drug–drug interactions and toxicities, which can occur frequently in patients at a high risk of invasive fungal infections who are often receiving multiple concomitant medications [8,9,10,11]. Thus, there is a true need for the development of new antifungals.

T-2307 (4-{3-[1-(3-{4-{amino(imino)methyl]phenoxy}propyl)piperidin-4-yl]propoxy} benzmide) is an investigational agent currently under evaluation and in development for the treatment of invasive fungal infections, including those caused by *Candida* species that are resistant to clinically approved antifungals, including azole- and echinocandin-resistant strains. The initial preclinical and early-stage clinical development of T-2307 has been conducted by FUJIFILM Toyama Chemical Co., Ltd., (Tokyo, Japan), which has recently assigned the rights for developing and marketing outside of Japan to Appili Therapeutics (Halifax, NS, Canada; ATI-2307) (http://fftc.fujifilm.co.jp/en/news/news191121e.html, accessed on 15 January, 2021). This review discusses the mechanism of action of T-2307, its spectrum of activity against various fungi, and what is known about its effectiveness in experimental models of invasive fungal infections. Key features of T-2307 are shown in Table 1.

## 2. Structure and Mechanism of Action

**Figure 1:**
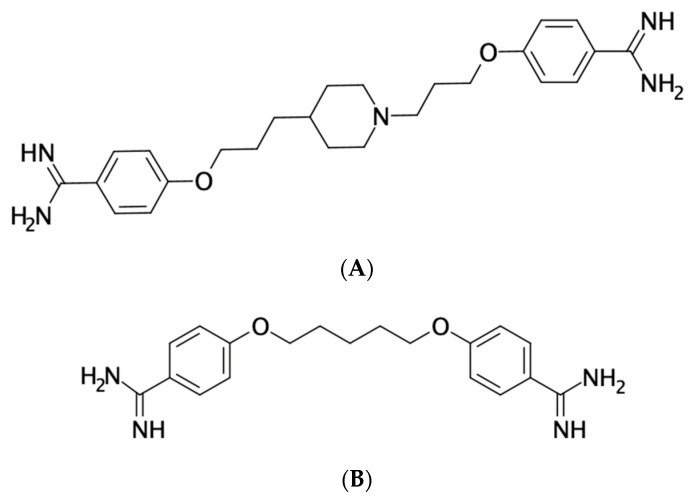
Chemical structures of the aromatic diamidines (**A**) T-2307 and (**B**) pentamidine.

Structurally, T-2307 is an aromatic diamidine, similar to pentamidine and furamidine (DB75) (Figure 1) [12]. Pentamidine can be used to treat pneumocystis, leishmaniais, and trypanosomiasis, and it is known that pentamidine and furamidine cause the collapse of the mitochondrial membrane potential within *Saccharomyces cerevisiae* [13,14,15]. A similar mechanism of action was suspected for T-2307 when it was observed that the degree of its activity against *Candida glabrata* was strongly influenced by the type and concentration of the carbon source in the growth medium. The trailing effect (i.e., incomplete inhibition of growth at concentrations above the minimum inhibitory concentration [MIC]) was reduced at lower glucose concentrations and was absent when the nonfermentative carbon source glycerol was used [14]. Similarly, *S. cerevisiae* cells grown in medium containing glycerol were also more sensitive to the effects of pentamidine and furamidine when compared to when they were grown in the presence of glucose. Similar results have also been reported for both T-2307 and pentamidine against *C. albicans* and *S. cerevisiae* when growth inhibition was compared between media containing dextrose and glycerol [13]. Interestingly, both the fermentative (in the presence of dextrose) and nonfermentative (in the presence of glycerol) growth of *Cryptococcus neoformans* was similarly inhibited by T-2307.

In order to assess the mechanism of action of T-2307 against yeast, Shibata et al. conducted a series of experiments [13]. To investigate the effects of T-2307 on the mitochondrial function in *S. cerevisiae* and *C. albicans*, the accumulation of the fluorescent dye MitoTracker Red CMXRos (MTR), a mitochondrial marker that concentrates in active mitochondria by membrane potential, was evaluated by microscopy. The presence of T-2307 at a concentration of 1 µM resulted in a less intense staining of mitochondria by MTR, indicating that the mitochondrial function had collapsed. These results were similar to those observed with carbonyl cyanide *m*-chlorophyenylhydrazone (CCCP), a protonophore and uncoupler of oxidative phosphorylation. In both *S. cerevisiae* and *C. albicans*, the number of whole cells with intense staining of the mitochondria decreased in the presence of T-2307 in a concentration-dependent fashion. In addition, a gradual and dose-dependent collapse of the mitochondrial membrane potential was also noted in mitochondria isolated from *S. cerevisiae* that were exposed to T-2307 or pentamidine. However, the T-2307 concentration required to cause a collapse in the membrane potential of at least 50% (30 µM) was markedly higher than the concentration required to inhibit the growth of the isolate (MIC 0.002 µM, or 0.001 µg/mL). With pentamidine, a reduction in the mitochondrial membrane potential of at least 50% occurred at a concentration of 5 µM, while the MIC against *S. cerevisiae* was reported to be 1.5 µM, or 0.5 µg/mL. The disconnect between the T-2307 concentration required to cause the collapse of the mitochondrial membrane potential and that needed to inhibit the growth of the organism (MIC) was postulated to be due to the intracellular accumulation of T-2307, as others have reported that this agent concentrates approximately 5000-fold within *C. albicans* cells from an extracellular medium via transporter mediated systems [16]. Subsequent work has indicated that T-2307 transport into *C. albicans* occurs through a high-affinity spermine and spermidine transport system that is regulated by Agp2, a plasma membrane protein involved in the update of L-carnitine and polyamines and other substrates in *S. cerevisiae* [17]. It is known that the uptake of polyamines, organic compounds having more than two amino groups, is dependent upon the mitochondrial membrane potential [18]. Interestingly, the activity of T-2307 against a *S. cerevisiae* strain with a Δ*agp2* mutation was markedly reduced when compared to the wild-type strain (MIC >8 µg/mL vs. 0.00012 µg/mL, respectively). The implications of this finding for the potential development of resistance to T-2307 are unknown.

The effect of T-2307 against rat liver mitochondria was also assessed [13]. No inhibitory effect on the rat mitochondrial membrane potential was observed with T-2307 at concentrations as high as 10 mM, which was at least 10 times higher than that observed with pentamidine, where collapse was observed at a concentration of 1 mM. Furthermore, the concentration of T-2307 within *C. albicans* cells was significantly higher than that observed in rat hepatocytes. Thus, T-2307 appears to have a greater selectivity for fungal rather than mammalian mitochondria and also concentrates to a greater degree within yeast cells when compared to mammalian cells.

To further assess the mechanism of action of T-2307 on the mitochondrial function, Yamashita et al. measured oxygen consumption in whole yeast cells [19]. Respiration was inhibited by T-2307 in a dose-dependent fashion, while respiration was not stimulated at any tested concentration (up to 1000 µM). Against intact yeast mitochondria, T-2307 caused a dose-dependent decrease in the rate of mitochondrial oxygen consumption in both the presence and absence of adenosine diphosphate (ADP). Similarly, potassium cyanide, a respiratory chain inhibitor, completely inhibited respiration under both basal and ADP-stimulated conditions, while oligomycin A, an ATP synthase inhibitor, did not affect the oxygen consumption rate in the absence of ADP. Thus, the oxygen consumption inhibition pattern observed with T-2307 indicates that this investigational agent inhibits the yeast mitochondrial respiratory chain. Further work from this study suggested that T-2307 induced mitochondrial dysfunction in yeast mainly through the inhibition of the respiratory chain enzyme complexes III and IV, and that ATP production was suppressed in a dose-dependent fashion, with the intracellular levels significantly decreased at T-2307 concentrations of ≥0.01 µM. However, against mitochondria isolated from a bovine heart, the T-2307 IC50 values were >3000 µM against all mitochondrial respiratory chain enzyme complexes. These results further demonstrate the selectivity of T-2307 against the yeast mitochondrial function.

## 3. In Vitro Spectrum of Activity

T-2307 has demonstrated an antifungal activity against several yeast species and some filamentous fungi, including isolates that are resistant to clinically approved azoles (Table 2). Against yeasts, it shows activity against *Candida* and *Cryptococcus* species, as well as *Malassezia furfur*. In an early screen for activity that included several ATCC and other reference strains of *Candida* and other yeasts and that utilized the Clinical and Laboratory Standards Institute (CLSI) broth microdilution methods with a prominent reduction in growth as the MIC endpoint, T-2307 demonstrated MICs ranging from 0.00025 µg/mL to 0.0039 µg/mL against *Candida* species, including four isolates of *C. albicans*, and single strains of *C. dublieniensis*, *C. glabrata*, *C. guilliermondii*, *C. krusei*, *C. parapsilosis*, and *C. tropicalis* each [12]. Against three *C. neoformans* isolates, the T-2307 MICs ranged from 0.0039 to 0.0078 µg/mL, and against a single isolate of *M. furfur* the MIC was 0.0313 µg/mL. Minimum fungicidal concentrations (MFC) were also determined, and the results were highly variable, with MFC values ranging from 0.0156 µg/mL to 0.0625 µg/mL against *C. neoformans*, but from 0.002 µg/mL to >64 µg/mL against different *Candida* species. Little to no activity was reported against the single isolates of *Saccharomyces cerevisiae* and *Trichosporon asahii* that were included in this study (MIC and MFC values of ≥64 µg/mL). Additional in vitro studies have reported that T-2307 maintains activity against echinocandin-resistant strains of both *C. albicans* and *C. glabrata*, with similar geometric mean (GM) MIC values reported for caspofungin-susceptible and -resistant isolates (T-2307 GM MIC range of 0.008 µg/mL to 0.0135 µg/mL against all studied isolates) [20,21]. Interestingly, a complete inhibition of growth was not achieved against *C. albicans* or *C. glabrata* isolates. Recently, in vitro activity has also been demonstrated against the emerging pathogen *C. auris*. In a study that used 23 *C. auris* isolates, including those available from the CDC FDA AR Bank (https://www.cdc.gov/drugresistance/resistance-bank/index.html, accessed on 15 January, 2021) and additional clinical isolates, the MICs for T-2307 ranged from ≤0.008 µg/mL to 0.015 µg/mL, and the activity of this investigational agent was maintained against strains that were resistant to fluconazole [22]. A separate study also reported activity against 15 clinical isolates of *C. gattii*, with MICs ranging from 0.0078 µg/mL to 0.0625 µg/mL [23]. In a time-kill analysis also performed as part of this work, T-2307 demonstrated a concentration-dependent fungicidal activity with reductions in colony-forming units of >3 log_10_ at concentrations four times as high as the MIC value or higher. A similar fungicidal activity was also noted with amphotericin B.

Each of the studies that has measured the activity of T-2307 against yeasts has used the prominent inhibition of growth as the MIC endpoint, similar to what is used for fluconazole, other azoles, and the echinocandins against yeast per the CLSI recommendations. Early studies with this investigational agent noted a trailing effect, defined as the partial inhibition of growth over an extended range of the concentrations above the MIC, when tested using the CLSI broth microdilution methods. The trailing effect observed against *C. glabrata* was attenuated when the glucose concentration in the growth medium was decreased to 0.1% or lower, and this effect was absent when a nonfermentative carbon source, glycerol, was used in place of glucose [14]. However, trailing was not attenuated when the pH of the medium was lowered. Similarly, the growth of *S. cerevisiae* cells was more sensitive to the inhibitory effects of furamidine or pentamidine when glycerol was the carbon source used in the growth medium rather than glucose. The clinical relevance of this is unknown, but it may influence in vitro susceptibility testing. Both the CLSI and European Union Committee on Antimicrobial Susceptibility Testing (EUCAST) antifungal susceptibility testing methods use glucose as the carbon source in their respective broth microdilution assays, rather than glycerol [24,25,26]. In addition, the glucose concentration in the CLSI method (0.2%) is lower than that used by EUCAST (2%). However, a lack of complete inhibition of growth has been observed against some isolates of different *Candida* species when the CLSI method has been utilized to measure the in vitro activity of this investigational agent [12,21,22]. It is currently unknown whether this may be strain-dependent.

T-2307 has also demonstrated an in vitro activity against some species of filamentous fungi, although the available data are limited. In a study that included several different genera of molds but with a limited number of isolates for each genus, T-2307 demonstrated activity when measured using a prominent reduction in growth as the endpoint rather than the complete inhibition of growth, as recommended by CLSI against filamentous fungi [12]. T-2307 activity was observed against *Aspergillus* species (MIC range: 0.0156 µg/mL to 4 µg/mL), *Fusarium solani* (0.125 µg/mL), and *Lichtheimia corymbifera* (0.5 µg/mL) [12]. However, a reduction to no activity was reported against single isolates of *Rhizopus oryzae* (>64 µg/mL)*, Scedosporium boydii* (4 µg/mL), *Mucor racemosus* (2 µg/mL), and *Trichophyton rubrum* (2 µg/mL). Fungicidal activity was limited, as the MFCs against most of the filamentous isolates ranged from 4 µg/mL to >64 µg/mL, with exceptions for the single *A. nidulans* (0.0313 µg/mL) and *A. niger* (0.0625 µg/mL) isolates that were included in the study.

## 4. In Vivo Effectiveness

Several studies have evaluated the in vivo effectiveness of T-2307 in experimental models of invasive candidiasis, and the in vitro activity observed for this agent has translated into efficacy against different *Candida* species. In a neutropenic murine model of systemic candidiasis caused by a wild-type strain of *C. albicans*, survival was significantly improved in a dose-dependent fashion, with 100% of mice that received doses of 0.02 mg/kg once daily surviving to the study endpoint [12]. The effective dose 50% (ED50) calculated in this study for T-2307 was 0.00755 mg/kg when compared to 0.128 mg/kg for micafungin and 0.0466 mg/kg for amphotericin B. Similarly, in a separate study, survival was also improved in a dose-dependent fashion in immunocompetent mice with systemic candidiasis caused by an echinocandin-resistant isolate that harbored an F641S amino acid substitution in Fks1p [21]. Kidney fungal burden, as measured by colony-forming units (CFU/g), was also significantly lower in each of the T-2307 dosage groups (>3 log_10_ CFU/g lower for T-2307 at doses of 0.75, 1.5, 3, and 6 mg/kg/day by subcutaneous injection) when compared to groups administered vehicle control or high-dose caspofungin (10 mg/kg/day by intraperitoneal injection). In a neutropenic murine model of invasive candidiasis caused by a *C. glabrata* strain harboring an Fks2p amino acid substitution (R1379S) known to result in reduced in vitro echinocandin susceptibility, the kidney fungal burden was also significantly reduced in mice treated with T-2307 at each of the above dosage levels when compared to the vehicle control and caspofungin administered at a dose of 1 mg/kg/day [20]. However, these reductions were less than 2 log_10_ CFU/g and were similar to the reductions observed with high-dose caspofungin (10 mg/kg/day). It is unknown if this difference in the fungal burden reduction between the *C. albicans* and *C. glabrata* models is due to real differences in the in vivo efficacy between these two species or due to a reduced effectiveness of T-2307 in the neutropenia setting, which was required in this model to establish the infection caused by *C. glabrata*. Previous work has reported differences in in vivo efficacy for anidulafungin and fluconazole between neutropenic murine and immunocompetent murine models of invasive candidiasis when the same strain of *C. albicans* was used to cause infection [27]. More recently, the in vivo efficacy of T-2307 has also been demonstrated against the emerging pathogen *C. auris*. In a neutropenic murine model in which infection was caused by a fluconazole-resistant isolate (fluconazole MIC >64 µg/mL, T-2307 MIC ≤ 0.008 µg/mL), treatment with T-2307 at 3 mg/kg/day resulted both in improvements in survival and reductions in the kidney fungal burden [22]. However, no reductions in colony-forming units were observed within the brain tissue of mice treated with this investigational agent.

Interestingly, another study reported that T-2307 was effective in reducing the ocular fungal burden in mice infected with a wild-type *C. albicans* (strain SC5314), both when treatment was initiated shortly after intravenous inoculation (i.e., 2 h—early-phase arm) and when delayed by 12 h (late-phase arm) [28]. In both the early-phase and late-phase arms, colony-forming units within the eyes and kidneys of mice treated with T-2307 at 4 mg/kg/day for three days were reduced by greater than 2 log_10_ when compared to untreated mice. Reductions in the kidney and ocular fungal burden were also reported in mice treated with liposomal amphotericin B (4 mg/kg/day IV) and fluconazole (75 mg/kg/day orally) in the early-phase and late-phase arms. The ocular T-2307 trough levels were also measured 24 h after a single dose in both infected and uninfected mice. T-2307 concentrations between infected and uninfected mice were similar (0.271 µg/mL vs. 0.245 µg/mL, respectively), and were well above the MIC measured against the strain used to cause infection (<0.001 µg/mL).

T-2307 has also shown in vivo efficacy in murine models of cryptococcosis. In a neutropenic model of systemic cryptococcosis caused by *C. neoformans*, dose-dependent improvements in survival were reported, with survival rates of 60%, 70%, and 100% at T-2307 doses of 0.1 mg/kg, 0.3 mg/kg, and 1 mg/kg administered once daily, respectively, and the ED50 was calculated to be 0.117 mg/kg [12]. Against pulmonary cryptococcosis caused by *C. gattii*, T-2307, at a dose of 2 mg/kg/day started 2 hours postinoculation, significantly reduced the fungal burden in both the lungs and brains of mice [23]. Similar results were also noted with amphotericin B (1 mg/kg/day) and high-dose fluconazole (160 mg/kg/day). However, only T-2307 was effective at reducing colony-forming unit counts when the start of therapy was delayed until day 14 postinfection. The authors also reported that T-2307 also prevented the progression of alveolar collapse caused by the proliferation of *C. gattii* observed within the lungs of mice not treated with this investigational agent. In vivo efficacy has also been reported against aspergillosis. In neutropenic mice infected with a wild-type *A. fumigatus* strain, survival was improved with T-2307, with survival rates of 90% at a dose of 1 mg/kg/day and 100% at a dose of 3 mg/kg/day [12].

## 5. Tolerability, Safety, and Clinical Data

Currently, no clinical data are available in the literature regarding the efficacy or safety of T-2307. A previous publication stated that this agent has successfully completed phase I studies without adverse effects [23], but these data are not currently available. In in vitro studies, as previously discussed, T-2307 has demonstrated a greater selectivity for the inhibition of yeast mitochondrial function when compared to that observed against rat and bovine mitochondria [13,19]. In addition, T-2307 has been reported to be well-tolerated in immunocompetent and neutropenic murine models of invasive fungal infections at doses of up to 6 mg/kg/day [12,14,20,21,22,23]. In contrast, pentamidine, which is structurally similar to T-2307, is known to cause numerous adverse effects/toxicities in both animals and humans, including serious endocrine/metabolic, cardiovascular, and renal toxicities when administered systemically [29,30,31,32]. T-2307 pharmacokinetic data are not available in the literature, and it is also unknown what formulations may be available for the treatment of patients.

## 6. Conclusions

T-2307 is an investigational agent that is currently in development for the treatment of invasive fungal infections. This agent has a novel mechanism of action and causes the collapse of the mitochondrial membrane potential, and studies have reported that this activity appears to be selective for fungi. T-2307 has both in vitro and in vivo activity against *Candida* species, including azole- and echinocandin-resistant strains, as well as both *Cryptococcus neoformans* and *C. gattii*. Activity has also been reported against some filamentous fungi, although the data are limited, and the complete inhibition of growth was absent or required elevated concentrations. Currently, clinical data demonstrating safety and efficacy in humans are not available.

## Figures and Tables

**Table 1:** Key features of T-2307.

**Mechanism of Action**	Causes the collapse of the mitochondrial membrane potential by targeting respiratory chain enzymatic complexes III and IV in the inner mitochondrial membrane of yeasts.
**Spectrum of Activity**	*Candida* species, including azole- and echinocandin-resistant isolates, *Cryptococcus neoformans* & *C. gattii.* Variable activity against *Aspergillus* species. Limited in vitro data against fungi suggests limited to no activity against *Fusarium*, *Scedosporium*, and the Mucorales.
**In vivo activity**	Efficacy in murine models of invasive candidiasis caused by *C. albicans*, *C. glabrata*, and *C. auris*; reductions in the ocular fungal burden caused by *C. albicans.* Efficacy in murine models of systemic aspergillosis due to *Aspergillus fumigatus* and cryptococcosis caused by *C. neoformans* and *C. gattii.*
**Pharmacokinetic and clinical data**	Not currently available in the literature.

**Table 2:** In vitro activity of T-2307. The results for which data are available for >10 isolates are shown. The endpoint that was used in all studies was a prominent inhibition of growth, and MICs are in µg/mL. MIC50 and MIC90 values represent MIC values at which 50% and 90% of the tested isolates were, respectively, inhibited.

Species	MIC Range	MIC50	MIC90
*Candida albicans*	0.00025–0.008	≤0.008	≤0.008
*Candida auris*	≤0.008–0.015	0.015	0.015
*Candida glabrata*	0.004–>4	≤0.008	≤0.008
*Candida guilliermondii*	0.001–0.004	0.002	0.004
*Candida krusei*	0.0005–0.002	0.001	0.002
*Candida tropicalis*	0.00025–0.0005	0.0005	0.0005
*Cryptococcus neoformans*	0.0039–0.06	0.015	0.03
*Cryptococcus gattii*	0.008–0.06	0.03	0.06
*Aspergillus fumigatus*	0.125–4	1	2
